# Linking Zoonosis Emergence to Farmland Invasion by Fluctuating Herbivores: Common Vole Populations and Tularemia Outbreaks in NW Spain

**DOI:** 10.3389/fvets.2021.698454

**Published:** 2021-08-12

**Authors:** Silvia Herrero-Cófreces, François Mougeot, Xavier Lambin, Juan José Luque-Larena

**Affiliations:** ^1^Dpto. Ciencias Agroforestales, Escuela Técnica Superior de Ingenierías Agrarias, Universidad de Valladolid, Palencia, Spain; ^2^Instituto Universitario de Investigación en Gestión Forestal Sostenible, Universidad de Valladolid, Palencia, Spain; ^3^Grupo de Gestión de Recursos Cinegéticos y Fauna Silvestre, Instituto de Investigación en Recursos Cinegéticos (IREC, CSIC-UCLM-JCCM), Ciudad Real, Spain; ^4^School of Biological Sciences, University of Aberdeen, Aberdeen, United Kingdom

**Keywords:** colonizing rodent, infectious pathogens, intensive agriculture, land-use changes, *Francisella tularensis*, *Microtus arvalis*

## Abstract

The expansion and intensification of agriculture are driving profound changes in ecosystems worldwide, favoring the (re)emergence of many human infectious diseases. Muroid rodents are a key host group for zoonotic infectious pathogens and frequently invade farming environments, promoting disease transmission and spillover. Understanding the role that fluctuating populations of farm dwelling rodents play in the epidemiology of zoonotic diseases is paramount to improve prevention schemes. Here, we review a decade of research on the colonization of farming environments in NW Spain by common voles (*Microtus arvalis*) and its public health impacts, specifically periodic tularemia outbreaks in humans. The spread of this colonizing rodent was analogous to an invasion process and was putatively triggered by the transformation and irrigation of agricultural habitats that created a novel terrestrial-aquatic interface. This irruptive rodent host is an effective amplifier for the *Francisella tularensis* bacterium during population outbreaks, and human tularemia episodes are tightly linked in time and space to periodic (cyclic) variations in vole abundance. Beyond the information accumulated to date, several key knowledge gaps about this pathogen-rodent epidemiological link remain unaddressed, namely (i) did colonizing vole introduce or amplified pre-existing *F. tularensis?* (ii) which features of the “*Francisella—Microtus*” relationship are crucial for the epidemiology of tularemia? (iii) how virulent and persistent *F. tularensis* infection is for voles under natural conditions? and (iv) where does the bacterium persist during inter-epizootics? Future research should focus on more integrated, community-based approaches in order to understand the details and dynamics of disease circulation in ecosystems colonized by highly fluctuating hosts.

## Introduction

Biological invasions and natural range shifts leading to colonization are processes that involve dispersal movements of species outside their historical distribution limits or from a point of introduction (1). The main difference between invasion and natural range shift lies in whether or not the species' arrival of species to new locations is directly mediated by humans, either deliberately or accidentally, as is the case of invasions (1). Yet, human activities can facilitate and promote shifts in species distribution by creating new opportunities and conditions that allow species to overcome factors limiting natural colonization and settlement (2). Human-driven distributional shifts are therefore conceptually very similar to invasive processes because they would not have happened without human intervention. Globalization, anthropogenic climate change and land-use changes thus represent major factors that alter species distribution and movement (3, 4).

Agricultural expansion and intensification have driven profound environmental changes worldwide, triggered by increased global food demand for a fast-growing human population (5). Agricultural practices directly affect habitat conditions and modify biotic communities, including host-pathogen systems (hereafter, we use the term “pathogen” to refer to both micro- and macroparasites) (6–8). The alteration of host-pathogen assemblages and their relative abundances can significantly modify the dynamics of infectious diseases, including zoonoses of global public health concern (3, 4). Different aspects of agricultural intensification, including land conversion and irrigation, have been associated with more than half of all 150 zoonotic infectious diseases that have emerged in the last 80 years (9). They include SARS, ebola, HIV, malaria, yellow fever, filariasis or trypanosomiasis (5, 9). Global trends in emerging infectious diseases reveal several concerning hot spots in Europe and North America (10), although zoonotic diseases remain most often associated with developing countries and poverty (11). Zoonotic risk is therefore a pressing global issue that requires major scientific attention and quantification worldwide.

Zoonoses emerging from wildlife populations represent a significant and growing threat to the human population (12). The largest number of zoonotic hosts is found among rodents (13, 14), and co-infections are a frequent phenomenon (15). The high adaptability of rodents has favored their success under human-driven landscape changes (14, 16, 17). Some rodent species with irruptive population dynamics (i.e., multi-annual “boom-bust” fluctuations in abundance) can be involved in the disease amplification, spillover and transmission to humans (16, 18). The role of native rodent hosts with highly fluctuating population has been critical in the emergence of many zoonoses worldwide, such as hantaviruses (19, 20), Lassa virus (21, 22), Andes virus (23), Lyme disease and tick-borne encephalitis (24). The challenge goes beyond the identification of a new rodent host but lies in understanding when changes in rodent communities resulting from invasions or range shift can lead to the emergence of novel pathogens in ecosystems, as in the case of *Yersinia pestis* and invasive *Rattus* sp. in Madagascar (25). The invasion of a new rodent host could lead to the establishment and emergence of the pathogen if all the necessary conditions, other than the presence of an effective amplifying host, were met prior to the invasion. The invasive rodent has to be a competent host for the pathogen, and the transmission between hosts and the pathogen reservoir must be efficient; asymptomatic hosts further facilitate the spread of the infection. Low minimum infection dose, broad host range and high survival of the pathogen in the environment all favor potential transmission. Environmental changes that increase the number and incidence of reservoirs and that enhance the probability of human contact are usually associated with emerging diseases. Anthropogenic causes including demographics, behavior, land use and health measures have been traditionally considered in epidemiology. The confluence of a new host, pathogen, reservoir, environmental and human features also play an important role here (26). From the current paradigm of a “One-Health” perspective, it is critical to understand the role that rodents invasions and fluctuations in abundance play in the epidemiology and dynamics of zoonotic diseases.

The common vole (*Microtus arvalis*) is the most widespread rodent in European agro-ecosystems (27, 28) and is characterized by its fast generation time, precocial reproduction, and cyclic fluctuations in abundance (29). Overabundances during population peak years periodically lead to economic losses (30), food and veterinary risks (29, 31, 32), cyclic zoonotic outbreaks (33), and recurring social conflicts (34). Many zoonotic agents are known to use common voles as hosts, from helminths (35) to viruses (36), along with a wide range of bacteria, protozoans and even fungi (37–40). This vole species can act as a vector of hantavirus (41), a reservoir of *Listeria, Babesia* and *Toxoplasma* (41, 42), and an intermediate host of *Echinococcus multilocularis* (43). Identifying the possible pool of pathogens, co-occurrence patterns and role of the host within the pathogen cycle is essential in order to uncover interspecific relationships that can modify the effect of those diseases on the host and the rest of the parasite community (44).

Here, we review a decade of research conducted on the biological interaction between the zoonotic bacterium *Francisella tularensis* and a colonizing rodent *Microtus arvalis* that massively invaded agricultural landscapes in NW Spain during the 1970–1990s (45). We use this case study to highlight how the irruptive population dynamics of a newly arrived rodent host are fundamental to our understanding of human tularemia outbreaks. The settlement of the fluctuating vole in NW Spain has triggered cascading effects via ecological interactions across several trophic levels, strongly influencing the dynamics of predators (46), ectoparasites (47, 48) and infectious pathogens (47–53). The biological consequences of this invasion and its repercussions on human public health have been the main focus of recent empirical work (33, 45–52, 54–59). In this review, we will first summarize the main research findings on the ecological and epidemiological interaction between *F. tularensis* and *M. arvalis* in intensively farmed landscapes from NW Spain and, subsequently, we will identify the main knowledge gaps of this pathogen-rodent dynamic system, which has so far officially affected more than 1,500 people in Spain since 1997 (Red Nacional de Vigilancia Epidemiológica [RENAVE]).

### Common Vole and Regional Colonization Process in NW Spain

**Figure 1:**
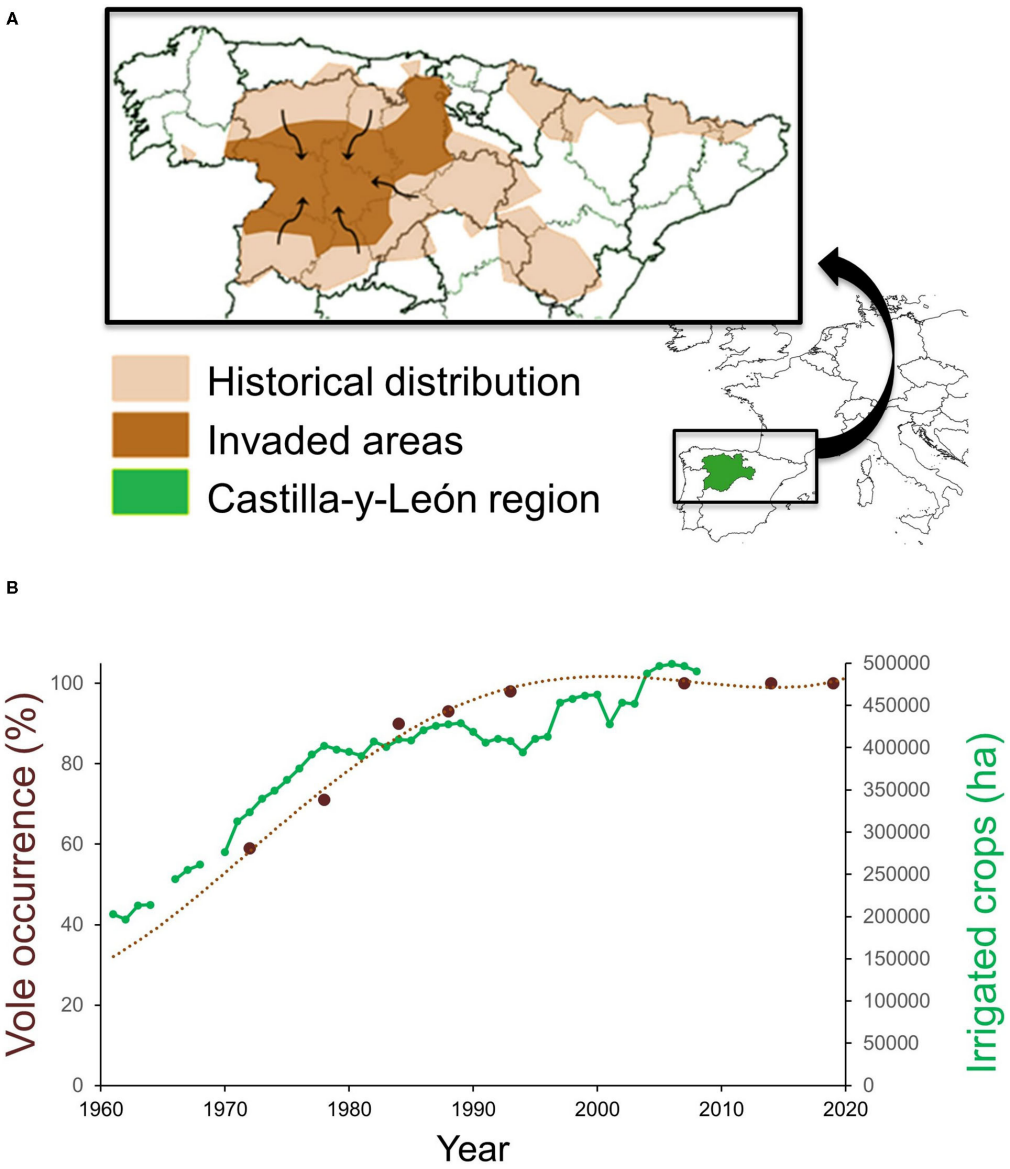
**(A)** Map of NW Spain showing the historical distribution and area invaded by common voles during the 1970–90s, and **(B)** temporal association between vole occurrence (percentage of agricultural counties with vole presence) and the extent of irrigated crops planted in the Castilla-y-León region, NW Spain. Adapted from Jareño et al. (55) and Luque-Larena et al. (59).

The common vole is a small herbivorous rodent with a strong preference for protein-rich plants and well-adapted to grasslands and steppe habitats (29). In NW Spain, common vole populations were historically restricted to mountainous habitats until the early 1970s (45) (Figure 1A). Populations in this historical range show density fluctuations with wide enough amplitude to influence rodent predator demography (60). The range expansion during the 1970–90s putatively emanated from surrounding wet mountainous habitat into drier lowland landscape (45), resulting in a geographical range expansion and colonization of ca. 5 million hectares of intensively cultivated agricultural areas (45, 59). Dramatic land-use changes had occurred prior to and during the colonization period: a network of irrigation infrastructure was built throughout the region, followed by a large increase in irrigated crop surface (Figure 1B) and the production of permanent fodder crops, especially alfalfa (55, 59). According to the possible dispersal pathways of Wilson et al. (2), the range expansion of the native common vole population followed an invasion pathway in which dispersal could be facilitated by the creation of corridors (habitats linked to the irrigation system network) interconnecting new suitable vole refuge habitats (permanent alfalfa crops). Aridity and summer droughts were likely to have been the main constraints preventing the common vole from colonizing xeric Mediterranean habitats (55). However, such natural limitation may have been lifted by increases in irrigation and protein-rich crop cultivation, providing suitable conditions for voles during the warmest and driest months of the year. Jareño et al. (55) determined that other concomitant changes, such as increased fall precipitation and higher winter temperatures, have also occurred but they may have played a less decisive role in the vole colonization. All available evidence supports that the massive range shift experienced by common vole populations in NW Spain was a human-driven event (55, 59).

### Common Vole and Human Tularemia Epidemics in NW Spain

**Figure 2:**
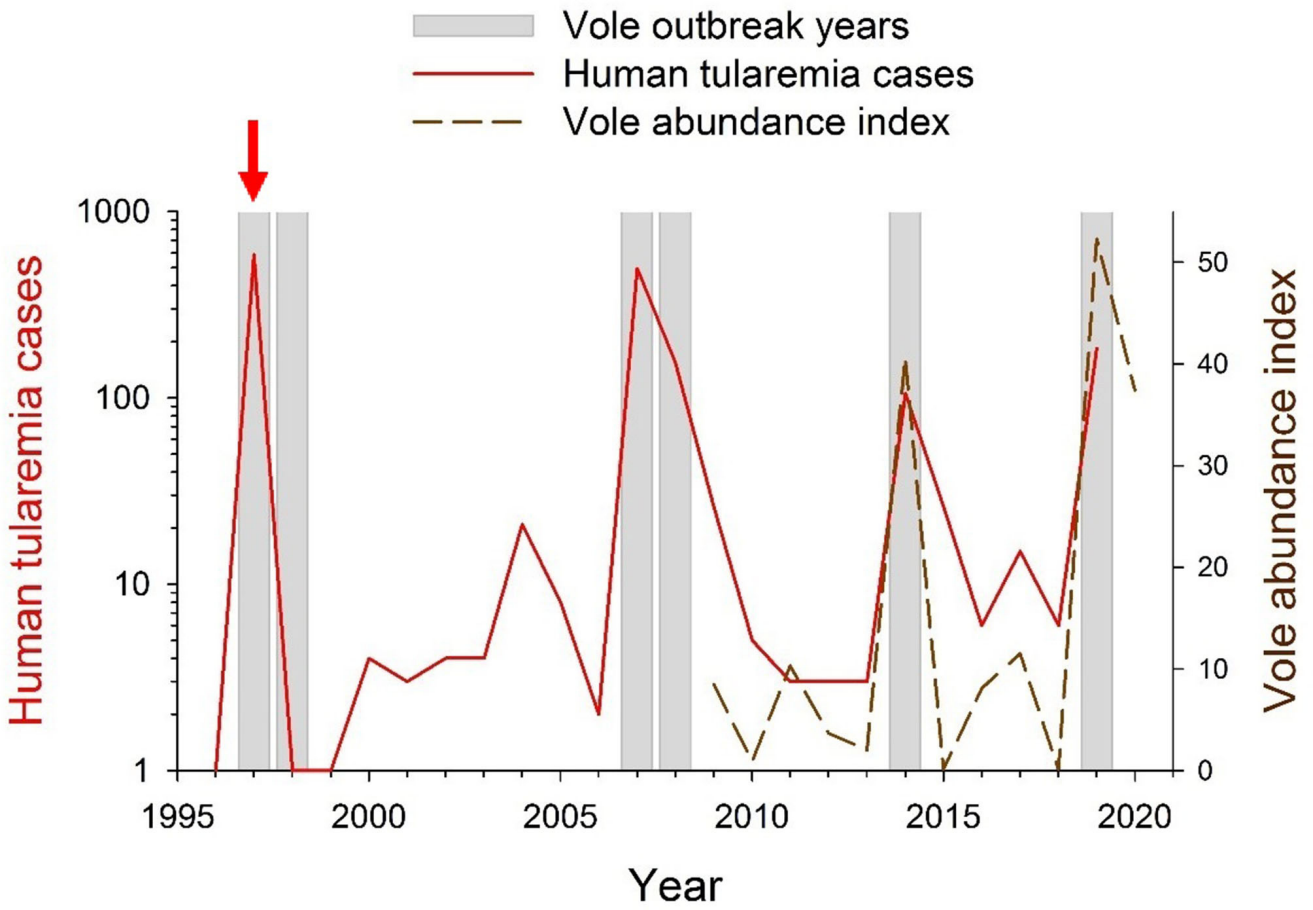
Yearly occurrence of vole and tularemia outbreaks in NW Spain between 1996 and 2020. During 1996–2008, vole outbreak years were identified by Luque-Larena et al. (45). During 2008–2020 vole outbreak years were identified based on common vole abundance indices obtained from live-trapping monitoring [(45, 48, 49), and unpublished data]. Data on yearly numbers of human tularemia cases (in red; log-transformed) were obtained from the Red Nacional de Vigilancia Epidemiológica (RENAVE; Instituto de Salud Carlos III, Madrid, Spain). The red vertical arrow indicates the year 1997, when tularemia became a notifiable disease in Spain.

Since farming landscapes were colonized by common vole populations, these have strongly fluctuated in abundance and region-wide outbreaks have occurred approximately every 5 years (45), with associated crop damages and socio-economical conflicts (34, 45). Vole outbreaks are recurrent in the study area (46) where local vole fluctuations have a 3-years periodicity since monitoring began in 2009 (46) (dashed line in Figure 2). Particularly relevant are the human public health concerns due to periodic tularemia outbreaks among the human population of the region that shares this agricultural area with common voles (34, 45).

Tularemia is an endemic zoonosis of the northern hemisphere caused by the highly infectious *Francisella tularensis*, classified as a Class A biothreat agent by the Center for Disease Control and Prevention (CDC) (61). There are four subspecies, but only two have human health implications: *F. tularensis* subsp. *tularensis* (type A) and *F. tularensis* subsp. *holarctica* (type B) (62). Type B is rarely fatal, but Type A is much more virulent for humans and, without treatment, it has an average mortality of 8% (up to 50% in some clinical forms) (63). There are five clinical manifestations (ulceroglandular, oculoglandular, oropharyngeal, pneumonic and typhoidal form) depending on the route of infection (skin inoculation, eye inoculation, ingestion, inhalation and undefined route, respectively) (62). This pathogen is a facultative intracellular Gram-negative bacterium known to be the zoonotic agent that infects the widest range of animal hosts (62). Lagomorphs and rodents have been long considered as main reservoirs and spreaders, while ticks, mosquitoes and flies could act as vectors (62, 64); the bacterium can also persist in watercourses hosted by the protozoan *Acanthamoeba castellanii* (65). Rodents have been associated with tularemia in different parts of the world since the disease was first reported (66, 67). In fact, the disease was initially named “plague-like disease of rodents” (68). Humans become infected by arthropods bites, by direct contact with infected animals, or by ingestion/inhalation of infective material (62, 64). Two different cycles have been described in Europe for tularemia: a terrestrial cycle, which includes lagomorphs, rodents and ectoparasite vectors such as ticks; and an aquatic cycle, which includes water, mosquitos, crayfish and semi-aquatic rodents such as beavers or muskrats (64, 66, 69). However, evidence from NW Spain suggests that both cycles are probably intertwined, as key terrestrial and aquatic agents effectively coexist in time and space (33).

Between 1992 and 2019, more than 20,000 human cases of type B tularemia were reported across Europe (64, 70, 71), mostly as discrete outbreak episodes spaced by interepizootic periods (54). In Spain, tularemia has been a notifiable disease since 1997, when the first large tularemia epidemic broke out in the Castilla-y-León region (Figure 1). Since then, three other large human outbreaks of tularemia have occurred in the region (Figure 2). The largest epidemics (with >500 confirmed human cases per outbreak) took place in 1997–1998 and 2007–2008 and were followed by relatively milder outbreaks during 2014 and 2019 (ca. 100 cases per outbreak). All human epidemic outbreaks recorded in NW Spain, without exception, closely coincide in time and space with large abundances of common voles in the environment (i.e., peak phase of their “boom-bust” cycles) [(55) and Figure 2]. When vole amplitudes did not reach a certain density threshold (as during the populations peaks of 2011 or 2017) there seems to be no spillover and no human epidemics were declared (Figure 2). However, further analyses should be done to confirm this hypothesis. Since the first record of the epidemic incidence of tularemia in 1997, approximately 1,500 clinical cases of human tularemia have been officially reported by the National Network of Epidemiologic Surveillance of Spain [Red Nacional de Vigilancia Epidemiológica (RENAVE), Instituto de Salud Carlos III, Madrid, Spain]. Most of these cases (97%) occurred in the intensive agricultural landscapes of Castilla-y-León, accumulating 92–100% of cases during epidemics (RENAVE). A serological survey carried out just before the first outbreak in 1997 revealed that the human exposure to *F. tularensis* in the region was very limited, with an average seroprevalence of 0.19% (72). This indicates that prior to common vole expansion the incidence of the disease in the region was negligible. Nevertheless, how seroprevalence has evolved among the human population since regular exposure to tularemia in 1997 and the length of seropositivity remains unaddressed.

### The Role of Vole Outbreaks in the Epidemiology of Tularemia

Recent studies have empirically demonstrated that fluctuating common vole populations in NW Spain contribute to amplifying *F. tularensis* in the environment, subsequently increasing the potential transmission routes and spillover to humans. For instance, during a large vole outbreak recorded between 2013 and 2015 (vole abundance peaked during summer 2014), the proportion of voles infected with *F. tularensis* directly increased with their abundance (58). This means that the more voles in the environment, the largest the population of zoonotic bacteria. Once vole numbers have collapsed, the pathogen was not detected among rodents in the study area (58). However, the sample size of tested voles during low-density periods (*n* = 35 from four trapping sessions) was small and provided limited evidence to establish whether *F. tularensis* infection is enzootic in voles or not. In NW Spain, tularemia epidemics in humans always occurred immediately after the number of infected voles has reached outbreak density, with no temporal delays (33, 54, 58).

In this case, tularemia epidemics are fuelled by vole numbers, which increase the pathogen pressure, the pathogen exposure to humans and the probability of infection in the ecosystem. The mean prevalence of *F. tularensis* among voles during a complete fluctuation can be as high as 33% at the maximum high-density phase (58). Thus, a direct density-dependent relationship between vole density and tularemia prevalence in voles has been thus established, supporting the hypothesis that common voles may play an important role in the amplification of the bacterium (33, 54, 58). This would increase pathogen pressure on voles and other competent hosts or vectors with which they share the agricultural landscape (i.e., ticks, fleas, hares). The high abundance of infected rodents during vole outbreaks also leads to a higher pathogen exposure to humans via direct contact or environmental contamination: voles drowning in irrigation canals contaminate water; voles dying in fields contaminate soils; and voles contributing to the cross-species transmission that eventually come in contact with humans (pets, livestock, crayfish, game species or invertebrate vectors) (50, 73). The use of anticoagulant rodenticides has been a common practice to attempt to control vole numbers during population outbreaks (45). It has been suggested that this practice could have worsened the epidemic situation in the past, concentrating the number of infected vole corpses in fields and thereby facilitating environmental contamination with infected material (50). Noteworthy, tularemia has been experimentally reported as both highly infective and lethal among voles (74), which underlies a significantly high probability of infection among voles.

Arthropod vectors are usually pointed as relevant agents involved in tularemia transmission elsewhere, including mosquitoes from the genera *Aedes, Culex* and *Anopheles*; ticks from of the genera *Ixodes* and *Dermacentor*; and flies from the genera *Chrysops, Tabanus* and *Chrysozona* (62). *Francisella tularensis* has been detected in other arthropods, but their vectoring role is still unclear (62). In NW Spain, common voles harbor mainly fleas (68% prevalence), and more rarely ticks (2% prevalence) (48). Human epidemics of several vector-borne zoonoses involving rodents typically show a one-year delayed pattern in other geographical areas (24, 75), including tularemia (76). However, in Spain, there is no evidence of any time delay between vole outbreaks and tularemia epidemics in humans (33) which, along with a very low mean prevalence of *F. tularensis* among fleas (collected from infected voles during the maximum density period), suggests a minor role of fleas in terms of tularemia circulation in our studied system (48).

**Figure 3:**
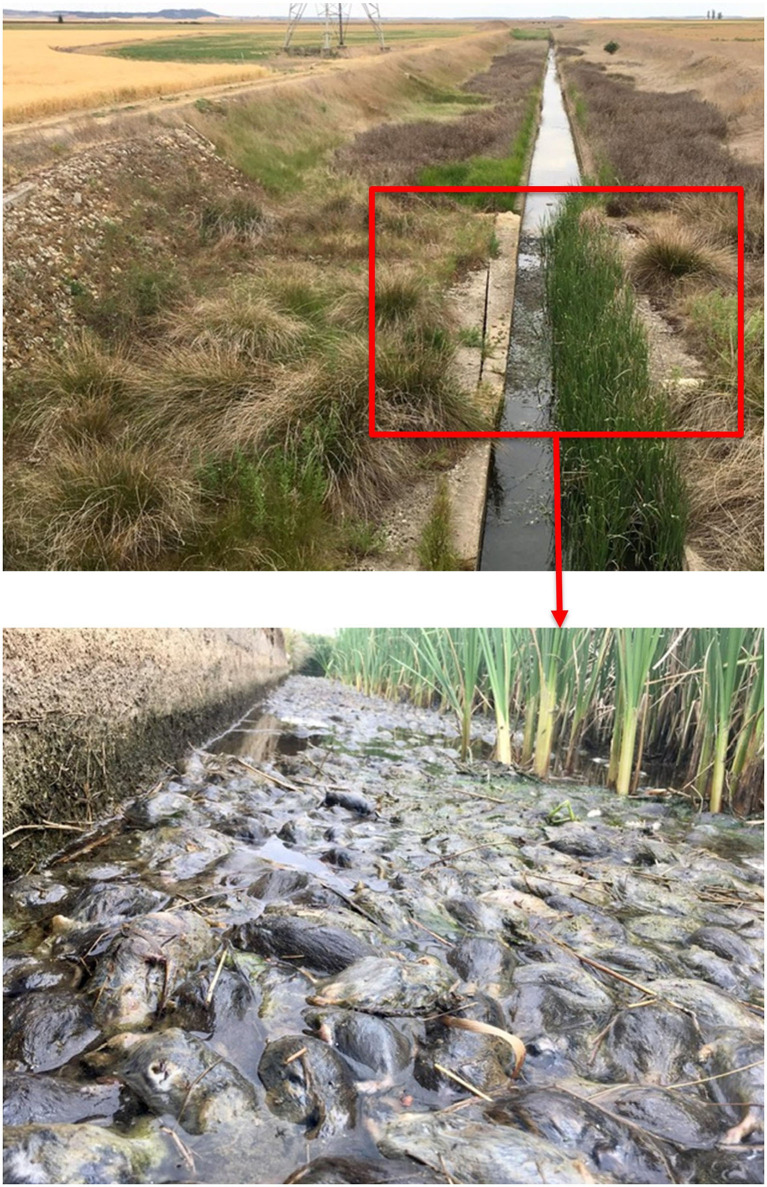
Common vole corpses decomposing in watercourses during a peak year (summer 2019). Photos by J.J Luque-Larena.

People could be infected with the bacterium during high-risk activities such as crop harvesting or manipulation of infected animals (i.e., harvested crayfish, hunted lagomorphs). Transmission may occur through skin lesions, contact with infective material or inhalation (50, 62). The route of infection typically determines the most common clinical manifestations in humans (62), which varies between outbreaks in the region (54). In the first epidemic (1997–1998), 93% of human cases were likely infected through direct contact with contaminated hares handled for consumption, causing a glandular form of tularemia. However, during subsequent epidemics, the underlying cause was probably the inhalation of contaminated dust or water (45–59% of infected people), causing a respiratory form of tularemia (77). The numerous vole corpses lying in the fields during the harvest and decomposing in watercourses during the irrigation period (Figure 3) could be the origin of the contaminated aerosols and therefore the proximate cause of disease spillover.

### Common Vole Outbreaks and Other Zoonoses in NW Spain

The common voles that invaded the farmed landscapes of NW Spain are good candidates to be zoonotic “hyper-reservoirs” (14) and may harbor and modulate other public health risks beyond tularemia. When screening common voles for zoonotic bacteria other than *F. tularensis* (i.e., *Anaplasma* spp., *Bartonella* spp., *Borrelia* spp., *Coxiella* spp., and *Rickettsia* spp.), only several species of the *Bartonella* genus were detected in the study area (49). *Bartonella* prevalence reached maximum values during vole outbreaks, when nearly 70% of the animals were infected by this zoonotic bacterium. Co-infection with different *Bartonella* spp. occured in more than half of the voles tested, and vole abundance, flea prevalence and coexisting mouse density influenced pathogen prevalence. Additionally, co-infection with *Bartonella* spp. and *F. tularensis* occurred in 13% of voles, reaching 24% of co-infection rate during the phase of maximum vole density (49). *Bartonella* is a highly interactive bacterium involved in positive and negative associations with other co-infectious pathogens in similar rodent systems with multiannual dynamics (44). The implications of this co-occurrence in the studied vole populations are, however, still unknown. Noticeably, some recent screening of the same vole populations has detected *Coxiella burnetii* (prevalence 12.2 %) and *Leishmania* spp. (1.2%) (53, 78). Sampling to evaluate disease dynamics in complex multi-species systems should always consider quantitative ecological perspectives. Regarding fleas parasitizing common voles in NW Spain, five species have been identified (79): *Nosopsyllus fasciatus, Leptopsylla taschenbergi, Ctenophthalmus apertus (subspecies gilcolladoi and apertus) C. baeticus* and *Rhadinopsylla beillardae*. The first three species were shared with other sympatric small mammals (79). In these fleas collected from voles, *Francisella* and *Bartonella* have been detected (48) but, conversely to what occurs in voles, this co-infection has not been found in their fleas (48). Recent research showed that *N. fasciatus* could hold a specific vectoring role of *Bartonella* spp. among rodent populations, in accordance with previous studies (80). Implications of flea species shared between the small mammal guild should be further investigated.

### Main Knowledge Gaps in the “*Francisella*–*Microtus*” System

Human-induced ecosystem modifications can lead to unexpected relationships between species that can result in the spread and spillover of zoonoses, as in common voles and tularemia in NW Spain (45, 54). This situation is in line with findings from a recent global meta-analysis showing that profound alterations due to agricultural intensification modify ecosystems and worsen zoonotic risks (81). The “*Francisella–Microtus*” case study has taught us the crucial role that a new rodent can play in disease dynamics when appropriate conditions are met, eventually triggering epidemics in humans. We have illustrated how the range shift of a native species at a regional scale is directly associated with the recent emergence of tularemia outbreaks among people. Our research provides convincing evidence that tularemia outbreaks in humans are closely linked with vole population outbreaks. However, there are a number of important knowledge gaps about the nature of the “*Francisella–Microtus*” epidemiological relationship under a dynamic host-density scenario, including the following key questions: (i) did colonizing voles introduce or amplified pre-existing *F. tularensis?*; (ii) what features of the “*Francisella–Microtus*” relationship are crucial for the epidemiology of tularemia?; (iii) what is the impact of *F. tularensis* infection on voles under natural conditions?; and *(iv)* where do bacterium populations remain when vole densities are very low (inter-epizootic period)?

### Key Knowledge Gap 1: Did Colonizing Vole Introduce or Amplify Pre-existing *F. tularensis*?

A first key issue is to determine if *F. tularensis* was co-introduced with the invading voles, or was it native but latent and amplified by *M. arvalis* in the region. In other words, we need to understand whether or not conditions for tularemia transmission were met prior to the introduction of the new rodent. Unfortunately, we lack data about tularemia prevalence before and during the range shift, hindering the evaluation of the real effect that vole colonization had on the system. The unspecific symptomatology of tularemia (62) means that it could have been diagnosed as “fever of unknown origin” (FUO) until the first severe epidemic broke out in 1997 (and tularemia became notifiable). Many zoonoses cause FUO in humans (82) and are underreported because they are misdiagnosed (83) or masked by another disease (82). Sampling the rodent population or conducting serological surveys of humans in the historical mountainous range of *Microtus arvalis* would be informative to address this issue.

Lagomorphs are considered competent hosts for the bacterium (73) and the Iberian hare *Lepus granatensis* is a traditional game species in the colonized area. Although hares have been directly linked to tularemia epidemics throughout Europe (73), no official epidemic event had been reported before the common vole colonization in NW Spain. If the condition for disease establishment were not met before the fluctuating vole became widespread, then it could have been introduced secondarily to its main competent host. Several pathways of introduction of this bacterium are plausible. One hypothesis is that tularemia was introduced by translocation of infected hares from other European countries (73) since *F. tularensis* genotype is the same as can be found in central and Western Europe (84) and hare translocations for hunting purposes have been frequent. Alternatively, migratory birds, in particular waterbirds, could have hosted subadult tick species involved in the tularemia cycle or transported the bacterium itself (73, 85). In the absence of evidence of tularemia outbreaks until the colonization of common voles, we favor the secondary introduction coinciding or following vole invasion rather than the amplification of a pre-existing pathogen.

### Key Knowledge Gap 2: What Features of the “*Francisella—Microtus*” Relationship Are Crucial for the Epidemiology of Tularemia?

An interesting issue is whether the parasite-host interaction or transmission cycles are qualitatively different in the colonized range compared to those in the native range. Serologically positive dogs and sheep have been detected within the historical vole distribution area (77), suggesting that the bacterium was present, although no human epidemic event have been notified there. Common vole populations have a continuous distribution in NW Spain (86). Since they are competent hosts for the bacterium, *F. tularensis* could be present to a greater or lesser extent over the whole species' range, although it has not been assessed yet. A relevant difference between invaded and native vole populations might be related to the amplitude of the abundance fluctuations. Vole populations fluctuate in the native range dominated by pastures (60), but with a much reduced amplitude (45, 46). Extremely high common vole densities (>1,000 voles /ha) are frequently reported in intensive agricultural landscape, and in alfalfa crops in particular (56). Thus, the boom-bust dynamic and high amplitude cycle may be a key feature to understand the tularemia epidemiology, especially when conditions allow for extremely high vole densities enhancing disease spillover potential. Another as yet unexplored possibility is that hosts may be more susceptible in the invaded than in the native range. This is the case of plague and rodents from different areas: disease resistance in Asian rodents is not present in North American ones, where microevolutionary resistance has not yet developed (87). Invasive and native rodents can show different response to the same disease. For example, European red squirrels are highly susceptible to squirrelpox virus introduced with the non-native gray squirrel, causing the decline of the native squirrel populations (88). Another possible explanation for the occurrence of epidemics in colonized areas but not in the traditional vole range could be differences in the transmission routes to humans, likely linked to differences in the agricultural work (crop harvesting, irrigation water) or wildlife consumption (crayfish, hare hunting) (77). The native and invaded common vole populations inhabit different agricultural systems (pastures and extensive cattle production vs. intensive irrigated cereal and alfalfa production), where tularemia transmission pathways or contact rates between people and infected hosts or vectors will likely be different. Furthermore, the level of human exposure to the bacterium in the historical range could be lower owing to the human population characteristics (scattered settlements and low population density). Finally, the lack or scarcity of a crucial vector, spreader or reservoir needed to complete the bacterium cycle could also result in a lower probability of infection of voles and the overall reduction of tularemia prevalence.

### Key Knowledge Gap 3: What Is the Impact of *F. tularensis* Infection on Voles Under Natural Conditions?

Given the virulence of the *F. tularensis*, infected animals are expected to quickly die (66). Voles experimentally infected with tularemia in the laboratory are known to shed large quantities of bacteria through feces and urine and to die rapidly (74), but very little is known about the effects of tularemia in voles inhabiting natural environments. Laboratory research has found differences in pathogenicity and disease duration between different *F. tularensis* genotypes (89). Furthermore, some classical research suggests the possibility of chronic tularemia nephritis in rodents (90). Vole species can recover from several viral and bacterial diseases within a few weeks while other diseases, such as vole tuberculosis and babesiosis, become chronic (91). Under a theoretical scenario where the main hosts (i.e., hares and voles) die fast after infection, tularemia persistence in the focal area could be compromised when low-density periods in host populations and unfavorable environmental conditions for the bacterium coincide. During peak phases and population crashes, most dead voles are probably infected by *F*. tularensis (50), yet some live-trapped *F. tularensis*-infected voles were asymptomatic and healthy in external appearance (58). Maybe these individuals will irretrievably die of tularemia at some point, but survive longer than under experimental conditions; or maybe their immune system could allow them to overcome the infection; or maybe they remain chronically infected and excrete the bacterium at low rates. In the absence of further serological, pathological and molecular evidence, the outcome of voles when facing the disease is yet unknown. Unveiling the duration of the disease infection and the mortality rate among reservoir populations would help characterize the course of tularemia epidemics. Intermediate levels of pathogen virulence may maximize transmission by balancing replication and host survival at increasing densities, and natural selection may favor the bacterium becoming deadlier as replication is not compromised (peak densities).

### Key Knowledge Gap 4: Where Does the Bacterium Persist During Inter-epizootics?

**Figure 4:**
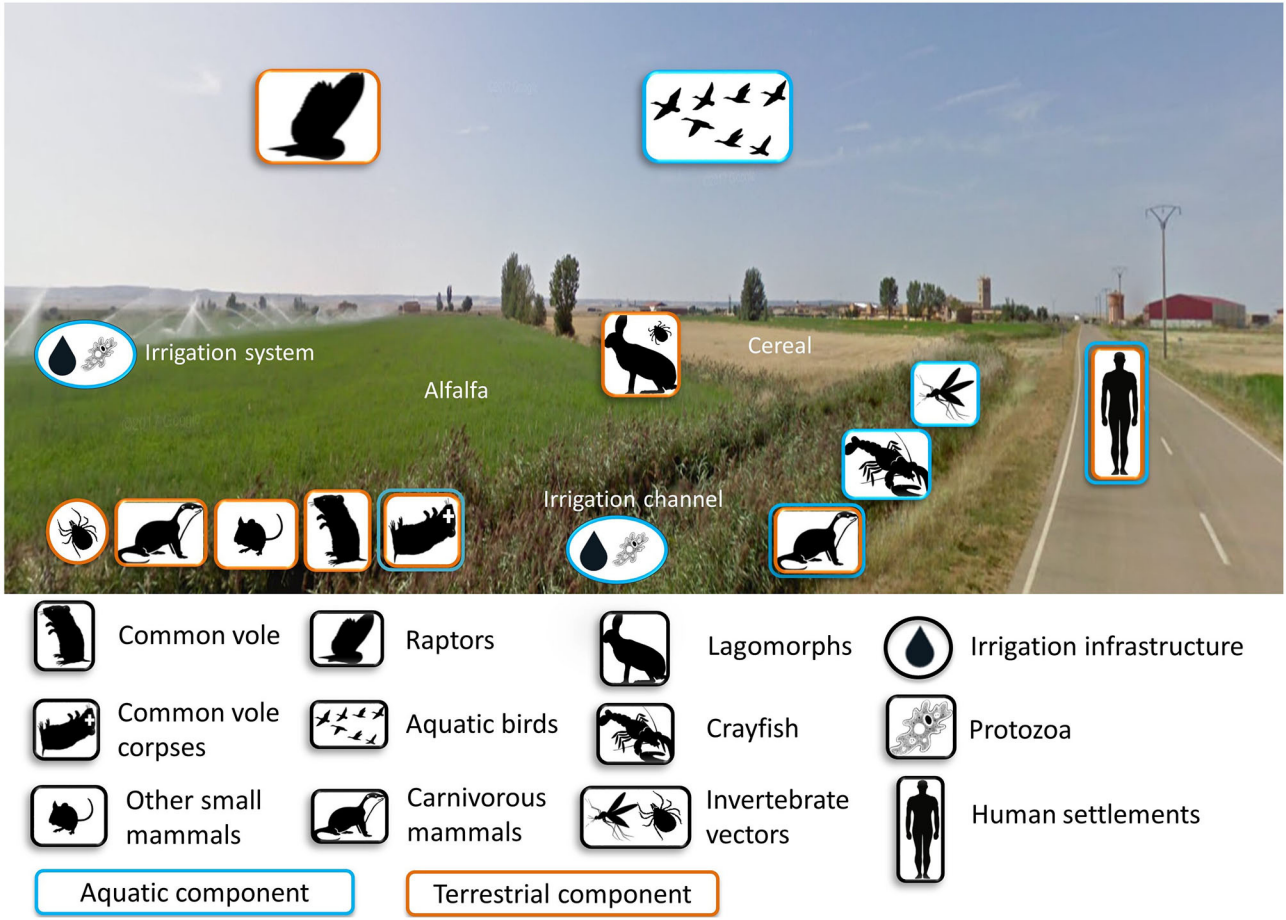
Components potentially involved in the cycle of tularemia either as amplifying, maintenance, spillover or dead-end hosts in intensive farming landscapes of NW Spain. Photo by GoogleMaps.

A high density of voles amplifies *F. tularensis* but voles are not always so abundant, even in the post-colonization era (46). Thus, a key knowledge gap is where the bacterium persists during inter-epizootics (i.e., between human tularemia outbreaks), and whether those refugia existed and may have been functional before vole colonization. The emergence of tularemia outbreaks is linked to voles, and bacterium survival may have been favored by the agricultural intensification. New aquatic habitats were created in the previously arid areas, thereby removing putatively limiting conditions for the persistence of *F. tularensis* or voles, and adding new potential reservoirs and hosts. In these farming landscapes, both terrestrial and aquatic components coexist within the same habitats (Figure 4), suggesting that the terrestrial and aquatic tularemia cycles act as a single unified cycle (33). A new terrestrial-aquatic interface arose with the consequent development of the typical fauna of this ecosystem that offers new possible hosts, reservoirs and vectors for the bacterium (i.e., waterbirds, aquatic protozoa, semiaquatic mammals and arthropods linked to aquatic habitats). Little is known about the role played by animals other than voles and lagomorphs in tularemia circulation in Spain. However, preliminary results have detected the bacterium in water, sediment and ticks from hares from the same habitats (51). Water and sediment are typical elements involved in the aquatic cycle, while ticks, hares and voles are typical hosts of terrestrial cycles. These findings support the hypothesis of a unified tularemia cycle in the region (33). In Europe, *F. tularensis* has been frequently detected in other small mammals (68), predators (92, 93) and arthropod vectors (94). It has also been isolated in samples from domestic cats (95) and migratory birds (96). These could be “dead-end” hosts, but we do not know to what extent they could be competent hosts amplifying the infection and potentially spreading it. Therefore, the circulation cycle may be more complex than initially expected. The determination of all biotic and abiotic elements and their role in tularemia circulation still remains partially unknown, especially during inter-epizootics periods. Many possible actors could be involved (Figure 4). And there are several non-exclusive scenarios, which can be tested by doing a longitudinal survey of tularemia prevalence in all the candidate reservoirs: (i) the bacterium remains in the environment (sediment, water); and/or (ii) the bacterium survives in other hosts (vole predators) that outlive the voles; and/or (iii) the bacterium persists in vectors (e.g., ticks, fleas) that may also survive from a vole outbreak to the next.

Determining the key reservoirs of *F. tularensis* is crucial to uncover how voles become infected during low-density phases. More research is needed to understand how voles get infected in the first place, before disease spillover, and for how long infected voles survive and spread the bacteria. Three main, non-exclusive, scenarios could occur. One possibility is that tularemia persists in surviving voles during inter-epizootics within small spatially limited refuges, which are hard to detect. Another alternative is that voles become recurrently reinfected by *F. tularensis* spill-over from alternative hosts, especially those species with larger lifespan, such as carnivores. Enzootic cycles involving lagomorphs and ticks are common in some tularemia endemic areas of the USA (97). In NW Spain, mean seroprevalence in lagomorphs during inter-epizootic periods (*n* = 515) varied between 0 and 14.6% (77), but their role remains understudied in depth. A third option would be that voles become reinfected from environmental reservoirs (Figure 4) as they widely spread through space. Contaminated water is a usual tularemia source in endemic areas of Northern and Eastern Europe (98). Once this first infection of voles has happened, high contact rates and transmission without lag in outbreaking vole populations may facilitate disease spread across the landscape. A second infection step from voles to the ecosystem would trigger the disease spillover during the common vole high-density phases, opening new transmission routes to humans. Future work should elucidate the mechanistic pathway explaining cyclical tularemia epidemics in NW Spain including how voles get infected before disease spillover, and how infected voles spread the bacteria to the system.

## Conclusions

Human-induced ecosystem modifications can favor unexpected relationships between species that result in the spread and spillover of zoonoses, as in common voles and tularemia in NW Spain (45, 54). The “*Francisella–Microtus*” case study shows us that the range shift of a native species at a regional scale can lead to the emergence of zoonotic epidemics when suitable conditions are met. Convincing evidence points that highly fluctuating dynamics of common voles are closely linked with tularemia outbreaks in humans. However, there are still some knowledge gaps that need to be investigated to further understand the epidemiology of tularemia in the focused area, such as the susceptibility and impact of *F. tularensis* infection on voles under natural conditions; uncovering all key vectors and reservoir hosts involved in the bacterium cycle; determining where the bacterium persists during inter-epizootics, and where it was before the first epidemic occurred. Future research should focus on more integral, community-based disease knowledge considering sympatric species, vole predators, ectoparasitic vectors, and alternative potential hosts and reservoirs. This will help to better comprehend the circulation of zoonoses infecting voles, forecasting new zoonotic disease emergencies, and elucidating possible effects of pathogens on vole populations.

## Author Contributions

SH-C drafted the manuscript. FM, XL, and JL-L critically revised the paper. All authors read and approved the final manuscript.

## Conflict of Interest

The authors declare that the research was conducted in the absence of any commercial or financial relationships that could be construed as a potential conflict of interest.

## Publisher's Note

All claims expressed in this article are solely those of the authors and do not necessarily represent those of their affiliated organizations, or those of the publisher, the editors and the reviewers. Any product that may be evaluated in this article, or claim that may be made by its manufacturer, is not guaranteed or endorsed by the publisher.
